# Highly Efficient Adsorption of Pb(II) by Magnesium-Modified Zeolite: Performance and Mechanisms

**DOI:** 10.3390/toxics14010085

**Published:** 2026-01-17

**Authors:** Yuting Yang, Xiong Wang, Sumra Siddique Abbasi, Bin Zhou, Qing Huang, Shujuan Zhang, Xinsheng Xiao, Hao Li, Huayi Chen, Yueming Hu

**Affiliations:** 1 School of Tropical Agriculture and Forestry, Hainan University, Haikou 570228, China; 2 Tropical Crops Genetic Resources Institute, Chinese Academy of Tropical Agricultural Sciences, Haikou 571101, China; 3 School of Environment Science and Engineering, Hainan University, Haikou 570228, China; 4 School of Architecture and Planning, Foshan University, Foshan 528000, China

**Keywords:** natural mineral, heavy metal, metal modification, precipitation method, ion exchange

## Abstract

In this study, magnesium-modified clinoptilolite (MZ) was successfully synthesized via precipitation and calcination to efficiently remove Pb(II) from aqueous solutions. The material was systematically characterized using BET, XRD, SEM-EDX, FT-IR, and XPS. Adsorption kinetics followed a pseudo-second-order model (R^2^ = 0.9956), with MZ removing over 70% of Pb(II) within the first 3 h. Isotherm data were best described by the Langmuir model (R^2^ = 0.9686), confirming monolayer chemical adsorption, with a maximum adsorption capacity (q_m_) of 1656 mg/g. Notably, MZ maintained high adsorption capacity across a pH range of 3.0~5.5, and its performance was largely unaffected by the presence of high concentrations of competing ions (0.1~1.0 M NaNO_3_). Mechanistic analysis revealed that the loaded MgO facilitates the chemical conversion of Pb(II) to hydroxycarbonate (Pb_3_(CO_3_)_2_(OH)_2_) via surface complexation, which constitutes the primary removal mechanism. These findings demonstrate that magnesium modification can transform natural zeolites into high-capacity, stable adsorbents, offering promising potential for the treatment of Pb(II)-contaminated water.

## 1. Introduction

Numerous industrial activities, such as lead ore mining, smelting, chemical manufacturing, agriculture, and electronic and semiconductor manufacturing, generate large amounts of lead (Pb)-containing wastewater [1,2]. Pb(II) is non-biodegradable and exhibits high biotoxicity [3]; it can readily accumulate in aquatic environments and enter the human body through the food chain, where long-term exposure may cause severe health hazards, including damage to the nervous system, renal dysfunction, and suppression of the hematopoietic system [4,5,6,7]. Therefore, the development of efficient, economical, and environmentally friendly remediation technologies for Pb-contaminated wastewater is of great importance.

At present, commonly used methods for Pb(II) contamination remediation include chemical precipitation, ion exchange, membrane filtration, and electrochemical treatment [8,9]. However, most existing remediation technologies suffer from high operational costs and excessive chemical reagent consumption [10,11], which has driven increasing efforts to explore alternative materials and approaches with higher efficiency and lower cost [12]. The adsorption method has been extensively studied and applied due to its operational simplicity, relatively low cost, and strong adaptability [3,8]. An ideal adsorbent should possess high adsorption capacity, rapid uptake kinetics, low cost, and environmental friendliness. Among various adsorbent materials, zeolites are regarded as highly promising heavy metal adsorbents owing to their abundant reserves, low cost, excellent ion-exchange capacity, and stable chemical properties [13,14,15,16].

Sprynskyy et al. [17] employed natural clinoptilolite as an adsorbent for the removal of Pb(II) from water. This zeolite was able to rapidly remove 25% of Pb(II) from solution within the initial 30 min, primarily through ion-exchange interactions between Pb(II) and the micropores on the zeolite surface. Karatas [18] and Perić et al. [19] further found that natural volcanic tuff zeolite and natural tuff zeolite could effectively remove Pb(II) from aqueous solutions with relatively low contamination levels. Zeolites possess a certain cation-exchange capacity [5,20,21] and exhibit a high affinity for Pb(II) [10], making them promising adsorbent materials. However, the adsorption capacity of natural zeolites for Pb(II) is relatively limited, and their adsorption kinetics are generally slow, which restricts their practical application in wastewater [22,23]. In the studies conducted by Sprynskyy et al. [17] and Karatas [18], the maximum adsorption capacities of natural zeolites were only 27.7 and 15.8 mg/g, respectively. To enhance the adsorption performance of zeolites, various modification strategies have been employed to functionalize these materials [10,23,24,25]. Metal modification can not only introduce new active sites but also alter the surface chemical properties of the material, thereby enhancing the affinity and selective adsorption of Pb(II) onto zeolites [13,25,26]. Yuan et al. [25] modified natural zeolite using magnetite and NaCl, producing a magnetic material (MMZ) with high magnetism and adsorption capacity, which increased the adsorption capacity of natural zeolite to 84 mg/g. In addition, Chen et al. [13] prepared a modified zeolite (NASO) using Al(NO_3_)_3_ and Na_2_SiO_3_ via stirring and heating; this material significantly enhanced the adsorption capacity for Pb(II) to 649 mg/g through chemical adsorption. Because magnesium (Mg) is relatively environmentally friendly and cost-effective, and because magnesium oxides/hydroxides can form stable surface complexes or precipitates with Pb(II), Mg-based modifiers have attracted considerable attention in recent years [26,27,28].

Therefore, in this study, natural clinoptilolite was selected as the raw material, and magnesium (Mg) was employed as a modifying agent to prepare a modified zeolite (MZ). The adsorption performance of MZ toward Pb(II) was systematically evaluated through kinetic experiments, isotherm studies, pH experiments, solid–liquid ratio tests, and ionic strength experiments. In addition, a combination of BET surface area analysis, scanning electron microscopy (SEM) and energy-dispersive X-ray spectroscopy (EDX), X-ray diffraction (XRD), Fourier transform infrared spectroscopy (FTIR), and X-ray photoelectron spectroscopy (XPS) was employed to comprehensively investigate the adsorption mechanisms of MZ. This study aims to provide theoretical support and technical references for the development of efficient, stable, and cost-effective materials for Pb(II) removal.

## 2. Materials and Methods

### 2.1. Materials

The raw clinoptilolite used in this study was obtained from Zhengzhou, Henan Province, China, with a particle size of 100 mesh. Magnesium chloride hexahydrate (MgCl_2_·6H_2_O), sodium hydroxide (NaOH), lead nitrate (Pb(NO_3_)_2_), and sodium nitrate (NaNO_3_) were analytical-grade reagents purchased from Xilong Scientific Co., Ltd in Shantou, Guangdong Province, China.

### 2.2. Synthesis of Magnesium-Modified Zeolite

The preparation method of magnesium-modified zeolite was adapted from Lu et al. [27]. Briefly, 5 g of pristine zeolite (Z) was immersed in 100 mL of a 1 mol/L MgCl_2_·6H_2_O solution. The suspension was magnetically stirred at 500 rpm for 1 h at room temperature. Subsequently, the pH of the mixture was adjusted to 10 using a 4 mol/L NaOH solution. The resulting precipitate was filtered, collected, and thoroughly washed with ultrapure water. The obtained solid was then calcined in a muffle furnace at a heating rate of 10 °C/min to 600 °C and maintained for 4 h, followed by re-grinding. Finally, the target adsorbent (MZ) was obtained after washing and drying.

### 2.3. Characterization

Z (0.25 g) or MZ (0.0125 g) was added to 50 mL of Pb(II) solution (1000 mg/L, pH 5.0) in a 100 mL conical flask and shaken at 25 °C and 180 rpm for 24 h to reach adsorption equilibrium. After filtration, the solid was freeze-dried, ground to a uniform powder, and stored in a desiccator prior to characterization. The specific surface areas and pore structural parameters of Z and MZ were determined by nitrogen adsorption-desorption isotherms using the multipoint BET method. The surface structure of absorbents were analyzed via scanning electron microscopy (SEM) coupled with energy dispersive X-ray analysis (EDX) using Zeiss Sigma 300 at 30 keV with background subtraction and a summation of 60 scans. X-ray diffraction (XRD) patterns were collected using Cu Kα radiation (λ = 1.5406 Å) at a power of 9 kW over a 2θ range of 3–130°, with a scanning rate of 10°/min. Fourier-transform infrared (FTIR) spectra of MZ were recorded in the range of 400~4000 cm^−1^ using a Bruker TENSOR 27 spectrometer. X-ray photoelectron spectroscopy (XPS) analysis was performed on a Thermo Fisher Scientific K-Alpha X spectrometer equipped with a monochromatic Al Kα source (hν = 1486.6 eV). Survey spectra (0~1200 eV) and high-resolution spectra were collected, and all binding energies were calibrated using the C 1s peak at 284.8 eV.

### 2.4. Batch Adsorption Experiments

Batch experiments were conducted in triplicate at 25 °C. Specifically, 50 mL centrifuge tubes containing the reaction mixtures were placed on a rotary shaker and agitated at 180 rpm. This study employed flame atomic absorption spectroscopy to determine lead ion concentrations in solutions before and after adsorption. Specifically, sample solutions before and after adsorption were appropriately diluted with 1% (*v*/*v*) nitric acid solution to ensure lead concentrations fell within the linear range of 0–5.0 mg/L prior to measurement. Analysis was performed using an atomic absorption spectrophotometer equipped with a lead hollow cathode lamp, measuring at the characteristic wavelength of 283.3 nm with an air-acetylene flame serving as the atomisation source. Key instrument parameters were set as follows: spectral bandwidth of 0.7 nm, lamp current of 5 mA, burner height of 8 mm, and acetylene flow rate of approximately 1.6 L/min. To ensure analytical accuracy and reliability, all determinations employed a 1% nitric acid solution as a laboratory blank.

The removal efficiency of Pb(II) was calculated as follows:

$$Adsorption\;rate\left(\%\right)=\frac{C_{0}-C_{e}}{C_{0}}\times 100$$

where C_0_ and C_e_ represent the initial and equilibrium Pb(II) concentrations (mg/L), respectively.

The adsorption capacity of the adsorbent was calculated using the following equation:

$$q_{e}=\frac{{(C}_{0}-C_{e})V}{m}$$

where q_e_ is the equilibrium adsorption capacity of Pb(II) (mg/g), C_0_ is the initial concentration of Pb(II) (mg/L), C_e_ is the equilibrium Pb(II) concentration in solution (mg/L), V is the volume of the Pb(II) solution (L), and m is the mass of the adsorbent (g).

#### 2.4.1. Adsorption Kinetics and Isotherm Experiments

The adsorption kinetics were first investigated. Specifically, 5 g/L of Z and 0.25 g/L of MZ were added to a 1000 mg/L Pb(II) solution at pH 5, and the mixtures were placed on a shaker. The solutions were agitated at 180 rpm for 1, 3, 6, 12, 18, 24, 36, and 48 h at room temperature (25 °C). After the designated shaking periods, the solutions were collected and filtered through a 0.45 μm membrane filter to remove any particulate matter. The Pb(II) concentration in the filtrates was subsequently measured. For the adsorption isotherm experiments, solutions with initial Pb(II) concentrations of 1, 2, 10, 20, 50, 100, 200, 500, and 1000 mg/L at pH 5 were prepared. Each solution was treated with 5 g/L of Z and 0.25 g/L of MZ and then placed on a shaker at 25 °C and 180 rpm for 24 h. After shaking, the solutions were filtered through a 0.45 μm membrane to remove Z and MZ particles. The Pb(II) concentrations in the resulting filtrates were measured to evaluate adsorption performance.

#### 2.4.2. Effect of Dose

To investigate the adsorption behavior of Z and MZ toward Pb(II) at different doses, the Pb(II) removal efficiency was evaluated over 24 h at pH 5 under varying solid–liquid ratios. For an initial Pb(II) concentration of 1000 mg/L, Z was added at 0.5, 1, 2.5, 5, and 10 g/L, whereas MZ was added at 0.05, 0.1, 0.25, 0.5, and 1 g/L. The mixtures were agitated on a shaker at 25 °C and 180 rpm for 24 h. After the designated contact time, the solutions were filtered through a 0.45 μm membrane to remove Z and MZ particles, and the Pb(II) concentrations in the filtrates were subsequently measured.

#### 2.4.3. Effect of pH

To investigate the adsorption behavior of Z and MZ toward Pb(II) at different pH levels, the initial pH of Pb(II) solutions was adjusted to evaluate the removal efficiency over a 24-h period. Pb(II) solutions with an initial concentration of 1000 mg/L were prepared at pH values of 3.0, 3.5, 4.0, 4.5, 5.0, and 5.5. Each solution was treated with 5 g/L of Z and 0.25 g/L of MZ and then agitated on a shaker at 25 °C and 180 rpm for 24 h. After the contact time, the solutions were filtered through a 0.45 μm membrane to remove Z and MZ particles, and the Pb(II) concentrations in the filtrates were subsequently measured.

#### 2.4.4. Effect of Coexisting Ion

To investigate the effect of coexisting ions on the adsorption of Pb(II) by Z and MZ, the Pb(II) removal efficiency was evaluated over 24 h by varying the initial ionic strength of the solution. Pb(II) solutions (1000 mg/L) were prepared at pH 5 with NaNO_3_ concentrations of 0.01, 0.1, and 1 mol/L. Each solution was treated with 5 g/L of Z and 0.25 g/L of MZ and then agitated on a shaker at 25 °C and 180 rpm for 24 h. After the contact time, the solutions were filtered through a 0.45 μm membrane to remove Z and MZ particles, and the Pb(II) concentrations in the filtrates were subsequently measured.

## 3. Results and Discussion

### 3.1. Kinetics

**Figure 1:**
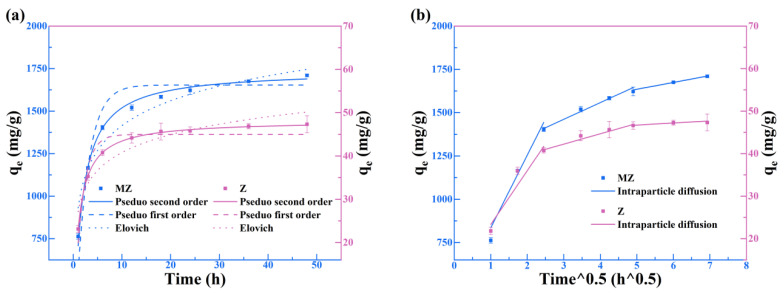
Effects of time on the Pb(II) adsorption. (**a**) Dynamics first-order, second-order, and elovich fitting models for Z and MZ; (**b**) Particle diffusion fitting model for Z and MZ.

The adsorption kinetics results are shown in Figure 1 (The error bars represent the standard deviation (SD) of three replicate experiments). The experiments indicate that the adsorption capacities of both Z and MZ increase rapidly with time before gradually reaching equilibrium. Notably, MZ reached adsorption equilibrium within 36 h, achieving an exceptionally high equilibrium adsorption capacity (q_e_ = 1740.25 mg/g). Although Z also reached equilibrium within 36 h, its equilibrium capacity (q_e_ = 48.17 mg/g) was far lower than that of MZ. This difference highlights that magnesium modification significantly enhances the adsorption performance of zeolite toward Pb(II). The adsorption data were fitted using pseudo-first-order (PFO), pseudo-second-order (PSO), and Elovich models, with the corresponding parameters summarized in Table 1. For both Z and MZ, the PSO model exhibited higher correlation coefficients (R^2^ = 0.9956 for MZ; R^2^ = 0.9997 for Z), indicating that the adsorption process is primarily governed by chemical adsorption [29,30].

To further understand the steps involved in the adsorption process, the data were analyzed using the intraparticle diffusion model. As shown in Figure 1b, both materials exhibit distinct multi-linear regions, suggesting that adsorption occurs through multiple sequential steps. As summarized in Table 2, the first adsorption stage exhibits the steepest slope, attributable to the rapid diffusion of Pb(II) to the external surface of the adsorbent or instantaneous surface adsorption. Notably, the intraparticle diffusion rate constant for MZ in this stage is much higher than that for Z, quantitatively confirming that magnesium modification introduces a large number of highly reactive sites on the zeolite surface. In the subsequent second and third stages, the adsorption slope gradually decreases (k_id1_ > k_id2_ > k_id3_), corresponding to the slow diffusion of Pb(II) into the internal pores and the approach to final adsorption equilibrium. Importantly, the fitted lines for all stages do not pass through the origin (intercept c ≠ 0), indicating that boundary layer diffusion (or film diffusion) is one of the rate-controlling steps in the adsorption process [31,32].

### 3.2. Isotherms

**Figure 2:**
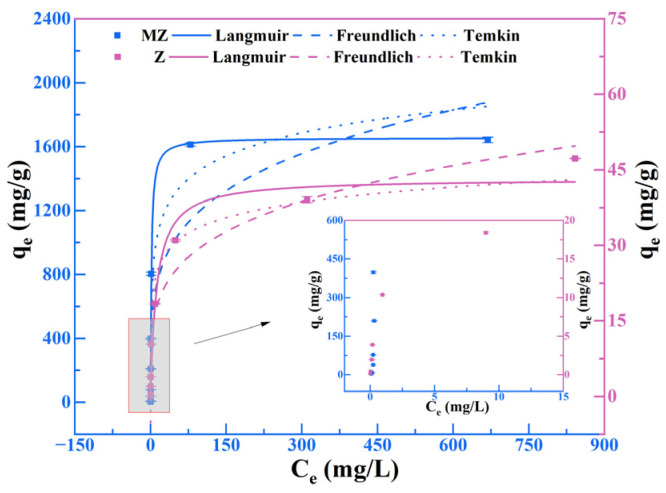
The effect of initial concentration on Pb(II) adsorption.

The adsorption isotherm results are presented in Figure 2 (The error bars represent the standard deviation (SD) of three replicate experiments), with the Langmuir, Freundlich, and Temkin model fitting parameters summarized in Table 3. For the pristine zeolite Z, all three models provided good fits (R^2^ = 0.9591, 0.9642, and 0.9636 for Langmuir, Freundlich, and Temkin, respectively), with the Freundlich model showing a slightly superior fit. The Freundlich model’s 1/*n* value is 0.24 (<1), indicating that the adsorbent exhibits a heterogeneous surface and undergoes positive adsorption [33]. However, the Freundlich constant K_F_ was relatively low (9.69 L/g), suggesting that the inherent adsorption capacity of Z is limited [26,34,35]. In contrast, the adsorption sites of magnesium-modified zeolite MZ appear to differ. Experimental data for MZ exhibited the highest correlation with the Langmuir model (R^2^ = 0.9686), significantly outperforming the Freundlich model (R^2^ = 0.8141) and the Temkin model (R^2^ = 0.9329). Calculations based on the Langmuir model indicate that MZ exhibits a theoretical maximum adsorption capacity (q_m_) for Pb(II) of 1656.04 mg/g. This represents approximately forty times that of Z and substantially exceeds most reported values for both natural zeolites and magnesium-modified zeolites (Table 4). This strongly suggests a more uniform energy distribution across the adsorption sites of MZ. Furthermore, the Langmuir constant K_L_ of MZ (0.578 L/mg) is markedly higher than that of Z (0.081 L/mg), indicating stronger affinity for Pb(II). These adsorption isotherm results demonstrate that magnesium modification significantly enhances the zeolite’s adsorption capacity for Pb(II) [2,36].

### 3.3. Effect of Dose, pH and Coexisting Ions

#### 3.3.1. Results of the Effect of Dose

**Figure 3:**
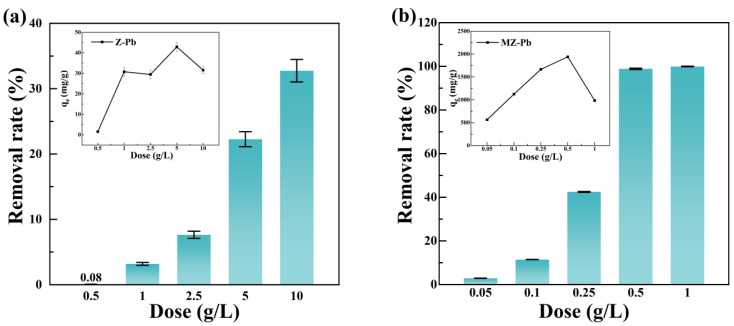
Effect of solid–liquid ratio on Pb(II) adsorption. (**a**) Effect of Z solid–liquid ratio; (**b**) Effect of MZ solid–liquid ratio.

Figure 3 (The error bars represent the standard deviation (SD) of three replicate experiments) illustrates the effect of adsorbent dosage on the Pb(II) removal performance of pristine zeolite Z and magnesium-modified zeolite MZ. For Z (Figure 3a), as the dosage increased from 0.5 to 10 g/L, the Pb(II) removal efficiency steadily rose from 0.08% to 32.75%, while the adsorption capacity increased from 1.52 to 31.54 mg/g. This concurrent increase indicates that the adsorption performance of Z is strongly limited by the number of available active sites on its surface. Increasing the dosage provides more adsorption sites [20], thereby enhancing the overall Pb(II) adsorption capacity of Z. However, even at high dosages, the adsorption capacity of Z remained relatively low.

In contrast, MZ exhibited highly efficient adsorption. As the dosage increased from 0.05 to 1 g/L, the Pb(II) removal efficiency rose sharply from 2.88% to 99.93%. Remarkably, at a dosage of 0.5 g/L, MZ had already achieved nearly complete Pb(II) removal, with the adsorption capacity reaching a peak of 1939.80 mg/g. Further increases in dosage led to a gradual decrease in the adsorption capacity. This trend reveals two key points: first, the surface-active sites of MZ exhibit extremely high affinity for Pb(II), allowing very small amounts of the adsorbent to effectively remove Pb(II) from solution; second, when MZ is in excess, the total Pb(II) available in solution becomes fixed, leading to a “dilution” effect on the adsorption capacity per unit mass of adsorbent.

#### 3.3.2. Results of the Effect of pH

**Figure 4:**
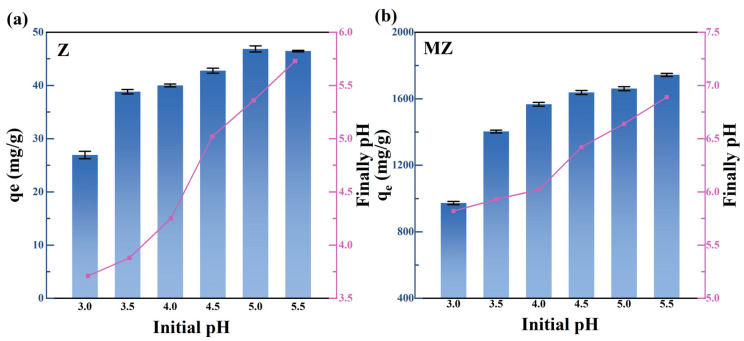
The effect of initial pH on Pb(II) adsorption by Z (**a**) and MZ (**b**).

The adsorption performance of pristine zeolite Z and magnesium-modified zeolite MZ at different initial pH values is shown in Figure 4 (The error bars represent the standard deviation (SD) of three replicate experiments). The adsorption of Pb(II) by Z exhibited strong pH dependence. At pH 3.0, the adsorption capacity of Z was very low (26.93 mg/g), but it increased significantly to 46.45 mg/g as the pH rose to 5.5. This behavior can be attributed to two main factors: first, at low pH, high concentrations of H^+^ ions compete with Pb(II) for the cation exchange sites on the zeolite surface, inhibiting Pb(II) adsorption; second, as the solution pH increases, the negative charge density on the zeolite surface gradually rises, enhancing Pb(II) adsorption via electrostatic attraction [2,38,42].

In contrast, MZ exhibited superior pH adaptability. Within the pH range of 3.5~5.5, MZ maintained high adsorption capacities (1403.06~1743.79 mg/g), consistently outperforming Z at all tested pH values. Even under strongly acidic conditions (pH 3.0), MZ retained a substantial adsorption capacity of 972.85 mg/g. This observation suggests that Pb(II) adsorption by MZ is not solely dependent on surface cation exchange or simple electrostatic interactions. We propose that the loaded magnesium species (e.g., MgO or Mg(OH)_2_) enable stronger and more specific surface complexation or precipitation reactions with Pb(II), which are less affected by competition from H^+^ ions, thereby ensuring effective Pb(II) adsorption even under acidic conditions [43,44].

#### 3.3.3. Effect of Coexisting Ions

**Figure 5:**
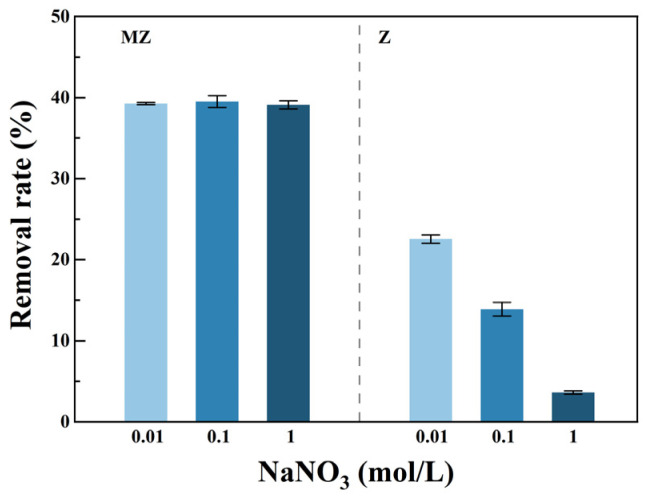
The effect of coexisting ions (NaNO_3_) on Pb(II) adsorption.

In practical water treatment, coexisting ions can affect the performance of adsorbents through competitive interactions. To evaluate the anti-interference capability of the materials, this study investigated the effect of background electrolyte ionic strength (represented by NaNO_3_ concentration) on the Pb(II) removal efficiency of pristine zeolite Z and magnesium-modified zeolite MZ, as shown in Figure 5 (The error bars represent the standard deviation (SD) of three replicate experiments). The adsorption performance of Z exhibited significant dependence on ionic strength. As the NaNO_3_ concentration increased from 0.01 to 1 mol/L, the Pb(II) removal efficiency of Z decreased sharply. This behavior is characteristic of ion-exchange or outer-layer electrostatic adsorption, where high concentrations of competing cations (Na^+^) can shield surface charges and compete with Pb(II), thereby inhibiting the adsorption process [45,46].

In contrast, MZ demonstrated excellent resistance to ionic interference. Across the same range of ionic strengths, the Pb(II) removal efficiency of MZ remained nearly constant, showing negligible sensitivity to changes in Na^+^ concentration. This difference indicates that the magnesium modification fundamentally alters the mechanism of Pb(II) adsorption on zeolite, such that adsorption on MZ is no longer dominated by outer-layer ion exchange or non-specific electrostatic interactions. Instead, adsorption likely occurs through inner-sphere surface complexation or precipitation, which are largely unaffected by the concentration of inert electrolytes in solution [47]. The ionic strength experiments reveal both the stability of Z and MZ under high ionic strength conditions and their intrinsic differences. While the adsorption of Z is strongly inhibited by coexisting ions, MZ exhibits robust anti-interference capability. Therefore, magnesium-modified zeolite may offer more stable and reliable performance for treating heavy metal-contaminated water containing high concentrations of background electrolytes.

### 3.4. Possible Adsorption Mechanism

#### 3.4.1. BET and SEM-EDX Results

**Figure 6:**
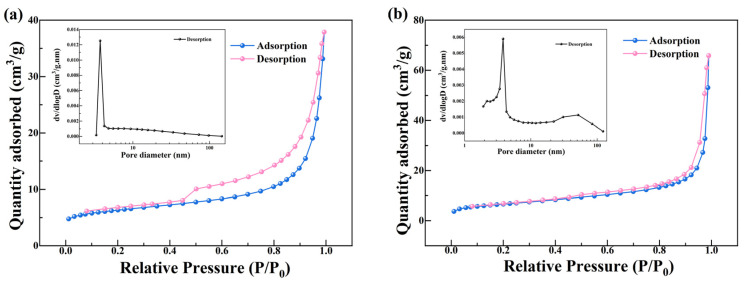
Absorption–desorption curve. (**a**) adsorption desorption curve of Z; (**b**) adsorption desorption curve of MZ.

Nitrogen adsorption-desorption measurements were performed to evaluate the specific surface area, pore volume, and pore size distribution of Z and MZ. As summarized in Figure 6, MZ exhibited a specific surface area of 23.66 m^2^/g, a total pore volume of 0.102 cm^3^/g, and an average pore diameter of 19.58 nm, whereas the corresponding values for Z were 22.90 m^2^/g, 0.059 cm^3^/g, and 13.76 nm, respectively. Both Z and MZ display typical type-IV adsorption-desorption isotherms accompanied by H3-type hysteresis loops, indicating that they are mesoporous materials with slit-shaped pore structures [48,49]. The overall similarity in isotherm shape before and after modification suggests that magnesium loading did not disrupt the intrinsic mesoporous framework of the zeolite. Notably, however, the nitrogen adsorption capacity of MZ (Figure 6b) at high relative pressures (P/P0 > 0.8) is significantly higher than that of Z (Figure 6a), which can be directly attributed to its markedly larger total pore volume (0.102 vs. 0.059 cm^3^/g). Additionally, the disappearance of hysteresis in MZ’s nitrogen isotherm fundamentally reflects how magnesium modification achieves zeolite pore structure reconstruction through the sequence: selective deposition of magnesium species—pore channel widening—enhanced connectivity. The narrow slit pores of the original zeolite Z caused high nitrogen desorption resistance, forming a pronounced hysteresis loop; whereas post-modification, MZ exhibits widened channels forming open mesopores with enhanced connectivity. This reduces the kinetic disparity between nitrogen adsorption and desorption, consequently diminishing the hysteresis phenomenon [50,51]. This result indicates that magnesium modification effectively expands the pore space of the zeolite, potentially facilitating enhanced mass transfer and providing additional accessible sites for Pb(II) adsorption.

A more detailed comparison of the pore size distribution curves (inset in Figure 6) provides clearer insight into the structural evolution induced by magnesium modification. Compared with Z, the pore size distribution of MZ shifts conspicuously toward larger pore diameters, with the average pore size increasing markedly from 13.76 nm to 19.58 nm. In addition, MZ exhibits a substantially stronger distribution intensity within the mesopore range of 10~50 nm. Meanwhile, its specific surface area shows only a marginal increase of approximately 3.3%. This distinctive combination—a pronounced increase in total pore volume and average pore size accompanied by only a slight increase in specific surface area—offers important clues regarding the modification mechanism. It suggests that magnesium species are likely deposited selectively on the internal surfaces of micropores or smaller mesopores, possibly in the form of nanoparticles or thin layers. Such deposition may partially block smaller pores or induce pore widening through localized restructuring, thereby merging adjacent pores into larger mesoporous channels. As a consequence, the material develops expanded pore space and higher pore accessibility without a significant change in overall surface area, which is consistent with the observed textural characteristics.

**Figure 7:**
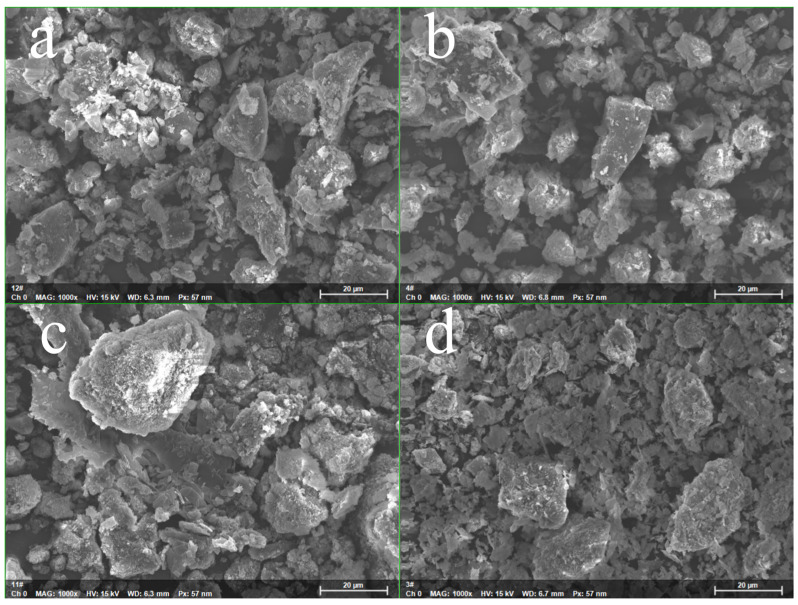

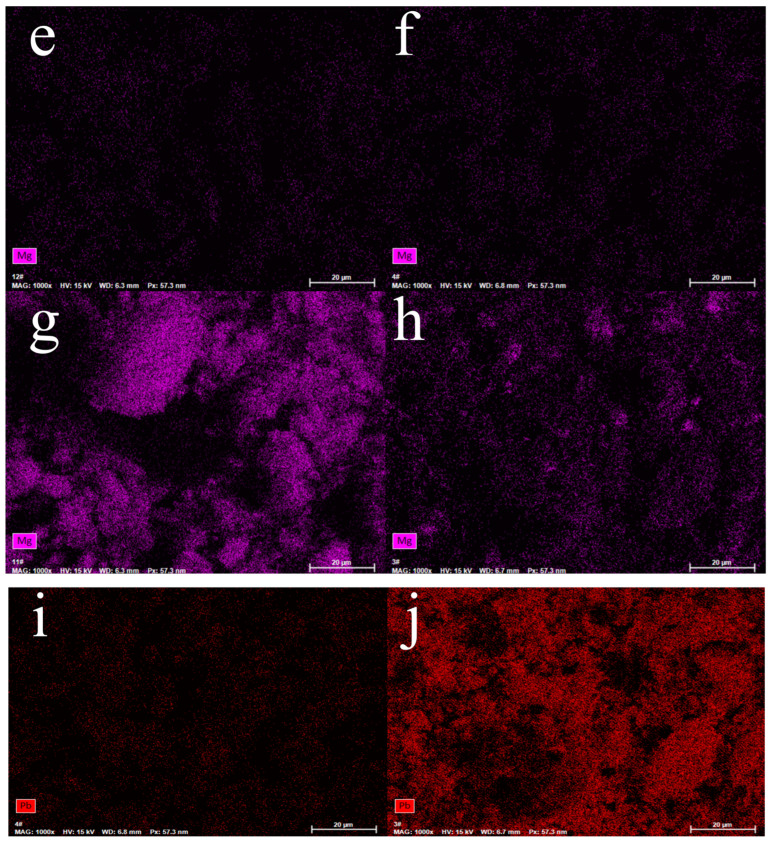
Scanning electron micrographs of Z and MZ before and after Pb adsorption. (**a**) SEM image of Z; (**b**) SEM image of Z-Pb; (**c**) SEM image of MZ; (**d**) SEM image of MZ-Pb; (**e**) EDX mapping of Mg in the aforementioned Z region; (**f**) EDX mapping of Mg in the aforementioned Z-Pb region; (**g**) EDX mapping of Mg in the aforementioned MZ region; (**h**) EDX mapping of Mg in the aforementioned MZ-Pb region; (**i**) EDX mapping of Pb in the aforementioned Z-Pb region; (**j**) EDX mapping of Pb in the aforementioned MZ-Pb region.

SEM-EDX was employed to characterize the surface morphology and elemental composition of Z and MZ (Figure 7 and Table S1). SEM images reveal that MZ exhibits a porous microstructure with irregular particle morphology, which is favorable for providing abundant accessible active sites and enhancing Pb(II) adsorption. The presence of a well-developed pore network in MZ further supports its high adsorption capacity. EDX analysis confirms the successful incorporation of Mg into the zeolite framework, with Mg contents consistent with theoretical values, indicating effective modification. After Pb(II) adsorption, a pronounced Pb signal is observed in the EDX spectra, demonstrating the strong Pb(II) uptake capability of MZ.

#### 3.4.2. XRD

**Figure 8:**
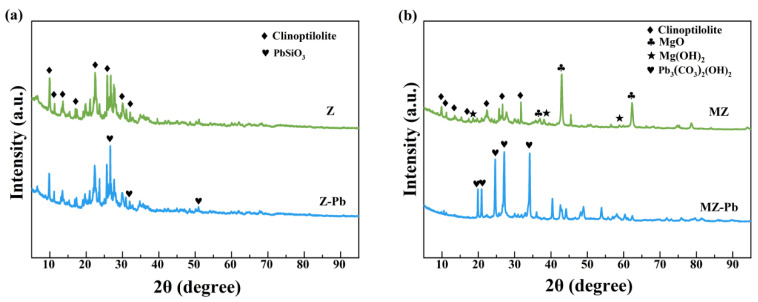
XRD phase analysis. (**a**) Z and Z-Pb; (**b**) MZ and MZ-Pb.

The XRD patterns of the Z and MZ before and after adsorption are presented in Figure 8. For the Pb(II)-adsorbed pristine zeolite (Z–Pb, Figure 8a), the main diffraction peaks match well with those of standard clinoptilolite (PDF#25-1349) [52,53], indicating that the adsorption process did not disrupt the primary framework structure of the zeolite [54,55]. The appearance of diffraction peaks corresponding to lead silicate (PbSiO_3_, PDF#74-1101) [56,57] suggests that Pb(II) adsorption on Z may involve interactions between Pb(II) and the silicate framework, resulting in the formation of sparingly soluble silicate phases; however, this mechanism provides only limited adsorption capacity [58].

In contrast, the XRD pattern of MZ (Figure 8b) provides strong evidence for the successful modification of the zeolite. Distinct diffraction peaks attributable to magnesium oxide (MgO, PDF#45-0946) [59,60] and magnesium hydroxide (Mg(OH)_2_, PDF#44-1482) [61,62] are clearly observed, confirming the formation and surface loading of crystalline magnesium species. Meanwhile, the characteristic peaks of clinoptilolite remain well preserved, indicating that the fundamental crystalline structure of the zeolite was not destroyed during the modification process. A comparison between the XRD patterns of MZ–Pb and Z–Pb reveals a fundamental difference in the Pb-containing phases formed after adsorption. Notably, the PbSiO_3_ phase observed in Z–Pb is absent in the MZ–Pb pattern. Instead, characteristic diffraction peaks of basic lead carbonate (Pb_2_(CO_3_)_2_(OH)_2_, PDF#13-0131) [63,64] are detected. This distinct phase transformation provides direct crystallographic evidence that Pb(II) adsorption on MZ proceeds via a mechanism fundamentally different from that on Z.

#### 3.4.3. FTIR

**Figure 9:**
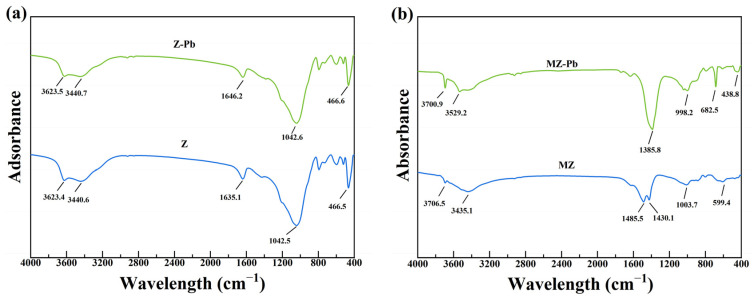
FTIR spectra of different samples. (**a**): Z and Z-Pb. (**b**): MZ and MZ-Pb.

Figure 9a presents the FTIR spectrum of Z. The broad band centered at approximately 3440 cm^−1^ indicates the presence of abundant hydroxyl groups on the sample surface. The absorption peak at 3623 cm^−1^ is attributed to the stretching vibration of Si–OH groups, while the band at 1646 cm^−1^ corresponds to the H–O–H bending vibration of adsorbed water molecules. The strong and broad band at 1042 cm^−1^ is characteristic of the asymmetric stretching vibrations of Si–O–Si and Si–O–Al bonds within the zeolite framework, whereas the band at 466.6 cm^−1^ arises from the bending vibrations of Si–O, Al–O, or Mg–O bonds in the framework [2,42,65]. These spectral features are consistent with the typical structural characteristics of clinoptilolite [66,67]. After magnesium modification, pronounced changes were observed in the FTIR spectrum of MZ. The Si–OH-related band at 3623 cm^−1^ almost disappeared, while the bands at 1042 cm^−1^ and 466 cm^−1^ exhibited noticeable shifts accompanied by reduced intensities, suggesting that Mg species partially occupied or altered surface sites originally associated with Si or Al atoms. Notably, a new band emerged at 3706 cm^−1^, which can be assigned to the stretching vibration of free or weakly coordinated Mg–OH groups, providing direct evidence for the successful loading of Mg-containing (hydr)oxide species on the zeolite surface [68,69,70]. In addition, the appearance of a doublet near 1485 and 1430 cm^−1^ is likely associated with surface carbonate or bicarbonate species (e.g., MgCO_3_), implying interactions between the loaded Mg species and atmospheric CO_2_ [71]. The newly observed band at 682 cm^−1^ can be attributed to lattice vibrations of Mg–O bonds, further confirming the successful incorporation of Mg phases [71].

The skeletal Si–O–Si, Si–OH, and surface hydroxyl peaks in the 3400–3800 cm^−1^ range for MZ and MZ-Pb before and after adsorption are nearly identical, with no new signals appearing. This indicates that ion exchange or inner-sphere complexation between Pb(II) and silicon hydroxyl groups is not the primary mechanism. In contrast, the sharp Mg–OH stretching peak at 3706 cm^−1^ exhibited a slight red shift to 3700.9 cm^−1^ with a marginally enhanced peak intensity. This indicates that the chemical environment of Mg–OH groups was disturbed by inner-sphere coordination of Pb(II). This displacement and peak enhancement phenomenon is consistent with reports in the literature concerning the formation of strong coordination interactions involving hydroxyl groups on the MgO surface [2,42]. Most notably, a distinct absorption shift at 1385 cm^−1^ is observed in the MZ-Pb spectrum. This band is typically associated with carbonate species (e.g., MgCO_3_) or vibrations of surface-bound nitrate ions, and may also originate from surface complexes or precipitated phases [26,72,73]. This shift further supports chemical interactions between Pb(II) and magnesium species on the MZ surface.

#### 3.4.4. XPS

**Figure 10:**
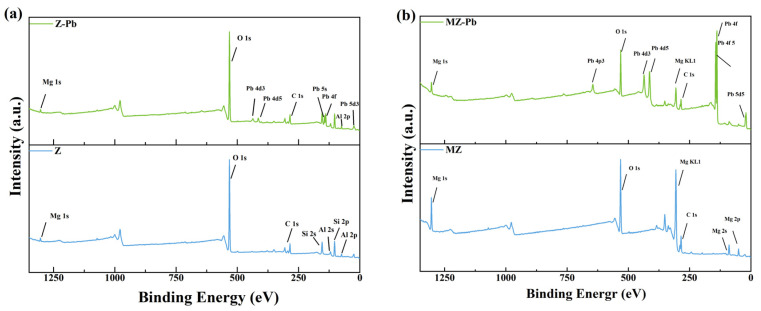
XPS spectra. (**a**) Z and Z-Pb; (**b**) MZ and MZ-Pb.

To further elucidate the interaction mechanisms between Pb(II) and Z as well as MZ, wide-scan X-ray photoelectron spectroscopy (XPS) analyses were carried out, and the results are presented in Figure 10. As shown in the survey spectrum of Z (Figure 10a), characteristic peaks corresponding to Al 2s, Al 2p, Si 2s, Si 2p, C 1s, and O 1s were detected, which is consistent with the elemental composition of aluminosilicate zeolite frameworks. The signal at 1034.30 eV, attributed to Mg 1s, was extremely weak, indicating that magnesium species were either absent or only present at trace levels in the pristine material. In contrast, the wide-scan spectrum of MZ (Figure 10b) exhibited several well-defined Mg-related signals, including Mg 2p (51.92 eV), Mg 2s (91.38 eV), Mg 1s (1306.38 eV), along with the characteristic Mg KL1 Auger peak at 305.30 eV [74]. Concurrently, the Al 2p signal originating from the zeolite framework exhibited a marked attenuation. This provides compelling evidence that magnesium has been successfully incorporated and enriched upon the zeolite surface, potentially partially coating or interacting with the framework to establish a new surface chemical environment dominated by magnesium, thereby achieving the modification objective. Concurrently, the intensity of the Si 2p signal was markedly attenuated. This phenomenon suggests that Mg-containing species were successfully introduced and preferentially enriched on the external surface of the zeolite, partially covering or replacing the original Si-based surface sites. These results confirm the successful surface modification of the zeolite by magnesium and validate the effectiveness of the modification strategy.

More importantly, XPS analysis provided direct evidence for element-specific chemical interactions occurring during Pb(II) adsorption, enabling the identification of adsorption mechanisms at the atomic scale. In the Z–Pb sample, the appearance of a Pb 4f signal at a binding energy of 139.16 eV confirms successful Pb(II) uptake. However, the binding energy position and intensity of the Mg 1s peak showed no appreciable change compared with pristine Z, indicating that the adsorption of Pb(II) onto Z was independent of magnesium species. This further implies that Pb(II) retention by Z is dominated by non-specific interactions such as physical adsorption or weak electrostatic attraction rather than Mg-mediated chemical bonding. By contrast, pronounced changes in the electronic structure were observed for the MZ–Pb sample. It is noteworthy that the Pb 4f peaks of Z-Pb and MZ-Pb exhibit distinct variations. By comparing the high-resolution Pb 4f spectra of Z-Pb and MZ-Pb (Figure S1), the changes in the chemical state of lead can be more precisely elucidated. In Z-Pb, the Pb 4f doublet peaks are located at 139.08 eV and 143.93 eV. In MZ-Pb, this characteristic peak pair systematically shifts towards lower binding energies by approximately 0.85 eV, now positioned at 138.23 eV and 143.08 eV, respectively. This pronounced negative binding energy shift indicates that the lead species immobilised on the MZ surface possesses a higher electron density [75]. Simultaneously, the Mg 2p peak displayed an evident increase in full width at half maximum (FWHM), and the Mg KL_1_ Auger peak became asymmetrically broadened. These spectral features indicate that the local chemical environment of Mg was significantly altered following Pb(II) adsorption, reflecting a direct chemical interaction between Pb(II) and Mg–O or Mg–OH moieties on the MZ surface. These binding energy shifts and peak broadening effects collectively demonstrate that Pb(II) was immobilized on MZ predominantly through strong chemical interactions, most likely via inner-sphere surface complexation and precipitation processes involving MgO or Mg(OH)_2_ phases [76]. The Mg-bearing functional groups acted as active centers that induced the nucleation and stabilization of Pb-containing solid phases, thereby transforming Pb(II) from a mobile ionic species into a chemically fixed form.

In summary, the enhanced Mg 1s signal intensity and the emergence of Mg 2p and Mg 2s peaks in the MZ survey spectrum unequivocally confirm the successful loading of magnesium species onto the zeolite surface. Furthermore, the pronounced positive shift of the Pb 4f binding energy in MZ–Pb provides compelling evidence for the chemisorption of Pb(II). At the atomic scale, these results demonstrate that Mg modification effectively creates new Mg–O-based reactive sites on the zeolite surface, and that Pb(II) immobilization at these sites proceeds mainly through the formation of strong chemical bonds via specific surface complexation mechanisms.

#### 3.4.5. Potential Mechanisms

The adsorption mechanisms of Pb(II) on Z and MZ were analysed by correlating adsorption behaviour with structural and surface characterisation results. BET analysis shows that the specific surface area of MZ (23.66 m^2^/g) is comparable to that of pristine Z (22.90 m^2^/g). In contrast, the total pore volume and average pore size of MZ increase markedly, from 0.059 to 0.102 cm^3^/g and from 13.76 to 19.58 nm, respectively. These results indicate that magnesium modification mainly alters the pore structure rather than increasing the accessible surface area, which is favourable for the diffusion and transport of Pb(II) within the particles. Therefore, the improved adsorption capacity of MZ cannot be explained solely by physical adsorption.

XRD patterns show that the clinoptilolite framework is preserved after modification, while additional diffraction peaks corresponding to MgO and Mg(OH)_2_ are observed in MZ, confirming the successful introduction of magnesium species. After Pb(II) adsorption, different solid phases are detected for Z and MZ. In Z–Pb, PbSiO_3_ is identified, whereas Pb_3_(CO_3_)_2_(OH)_2_ is detected in MZ–Pb. This difference suggests that magnesium modification changes the dominant Pb(II) removal pathway, promoting the formation of new lead-containing phases on MZ.

FTIR spectra further support this interpretation. For Z, the characteristic bands associated with framework Si–OH (3623 cm^−1^) and Si–O–Si (1042 cm^−1^) exhibit no obvious changes after Pb(II) adsorption, implying limited involvement of silanol groups in Pb binding. In contrast, the Mg–OH stretching band in MZ shifts from 3706 to 3700.9 cm^−1^ after adsorption, accompanied by changes in band intensity, indicating an interaction between Pb(II) and surface Mg–OH groups. In addition, the appearance of a band at 1385 cm^−1^, assigned to carbonate-related vibrations, is consistent with the formation of lead carbonate or hydroxycarbonate species, in agreement with the XRD results.

XPS analysis provides further evidence for the altered chemical environment of Pb in MZ. Compared with Z–Pb, the Pb 4f binding energy in MZ–Pb shifts by approximately 0.85 eV, together with an increased signal intensity. Meanwhile, changes in the Mg 2p peak width and the Mg KL_1_ Auger feature suggest that the chemical state of surface magnesium species is affected after Pb(II) adsorption. These observations indicate that Pb(II) interacts strongly with Mg–O sites on MZ, likely through inner-sphere complexation followed by the formation of stable lead-containing phases.

## 4. Conclusions

This study demonstrates that MZ is an effective adsorbent for the removal of Pb(II) from aqueous solutions. Kinetic analysis indicates that the adsorption process is best described by a pseudo-second-order model (R^2^ = 0.9956), suggesting that chemical adsorption is the primary rate-limiting step. Within the studied concentration range, isotherm data most accurately conformed to the Langmuir model (R^2^ = 0.9686), confirming the homogeneous surface of MZ and its maximum adsorption capacity of 1656 mg/g. Experiments assessing the effect of pH showed that MZ maintained high adsorption capacities across a broad pH range of 3.0–5.5. Notably, its adsorption performance remained largely unaffected even in the presence of high concentrations of competing Na^+^ ions (0.1–1.0 mol/L NaNO_3_). Mechanistic characterization revealed that the successfully loaded MgO and Mg(OH)_2_ facilitate the conversion of Pb(II) to Pb_3_(CO_3_)_2_(OH)_2_ via surface complexation, which constitutes the primary mechanism for Pb(II) removal by MZ. These findings demonstrate that this modification strategy transforms natural zeolite into an adsorbent with ultra-high capacity and excellent environmental stability, highlighting its significant potential for the treatment of lead-contaminated wastewater. Future research should prioritize industrial implementation by optimizing large-scale continuous precipitation–calcination processes to produce mechanically robust granular MZ suitable for fixed- or fluidized-bed systems. Pilot-scale studies in real lead-containing wastewaters are required to evaluate long-term operational stability and selectivity under multicomponent conditions, together with the development of mild regeneration and lead recovery strategies to enable adsorbent reuse and cost reduction. Finally, integrated life cycle assessment and techno-economic analysis should be conducted to assess the environmental impact and economic feasibility of MZ from production to application.

## Figures and Tables

**Table 1:** Parameters for the first-order kinetic model, second-order kinetic model and elovich model of Pb(II) adsorption on Z and MZ.

Adsorption Kinetic	Parameters	Z	MZ
Pseudo-first-order kinetic	R^2^	0.9198	0.9396
	k_1_ (h^−1^)	0.5859 ± 0.0799	0.4091 ± 0.0373
	q_e_ (mg/g)	44.9679 ± 1.1427	1653.5168 ± 30.3926
Pseudo-second-order kinetic	R^2^	0.9997	0.9956
	K_2_ (g/mg h)	0.0191 ± 0.0002	0.0004 ± 0.0001
	q_e_ (mg/g)	48.1736 ± 0.0839	1740.2486 ± 10.3087
Elovich	R^2^	0.9060	0.9666
	α (mg/g h)	555.7192 ± 442.3289	17,207.0277 ± 8293.7080
	β (g/mg)	0.1676 ± 0.0226	0.0047 ± 3.6678 × 10^−4^

**Table 2:** Parameters for the intraparticle diffusion model of Pb(II) adsorption on Z and MZ.

Intra-Particle Diffusion Steps	Parameters	Z	MZ
Step 1	R^2^	0.9378	0.9561
	k_id1_	12.6786 ± 3.2659	419.6162 ± 89.9102
	C_1_	10.6514 ± 6.3763	416.024 ± 165.0922
Step 2	R^2^	0.9794	0.9883
	k_id2_	2.4286 ± 0.24880	96.5521 ± 7.4349
	C_2_	34.9528 ± 0.8791	1172.1019 ± 27.5624
Step 3	R^2^	0.8879	0.9905
	k_id3_	0.4928 ± 0.1751	39.3592 ± 3.8604
	C_3_	44.2463 ± 1.0109	1438.4051 ± 23.9340

**Table 3:** Parameters of Langmuir and Freundlich models for the adsorption of Pb(II) on Z and MZ.

Model	Parameters	Z	MZ
Langmuir model	R^2^	0.9591	0.9686
	q_m_ (mg/g)	43.2181 ± 2.8819	1656.0373 ± 92.4428
	K_L_ (L/mg)	0.0809 ± 0.0294	0.5781 ± 0.1393
Freundlich model	R^2^	0.9642	0.8141
	1/n	0.2428 ± 0.0274	0.2334 ± 0.0536
	K_F_ (L/g)	9.6907 ± 1.5448	410.9136 ± 123.8827
Temkin	R^2^	0.9636	0.9329
	A (mg/L)	22.0043 ± 8.7963	11.2040 ± 4.4479
	B	4.3804 ± 0.3218	207.5263 ± 21.0387

**Table 4:** Adsorption capacities of different adsorbents on Pb(II).

Adsorbent	Modifying Agent	pH	Initial Concentration (mg/L)	Solid– Liquid Ratio (g/L)	qm (mg/g)	K_L_ (L/mg)	K_d_ (L/g)	References
CFA-Zeolite	NaCl	/	1000	/	624	0.230	143.06	[1]
Zeolite-NaX	NaOH	5	100	/	322	0.227	153.59	[37]
Zeolite-NZVI	FeCl_3_·6H_2_O	6	100	0.50	85	0.490	41.65	[5]
Zeolite-NASO	Al(NO_3_)_3_, Na_2_SiO_3_	5	1000	1.00	649	0.900	584.10	[13]
Nano-Zeolite-NaX	NaOH	/	1000	/	73	0.230	16.79	[20]
Zeolite-NaX	NaOH	5	1000	0.30	677	0.227	153.68	[38]
Zeolite-NaX(C)	NaOH	5	1000	0.30	322	1.178	379.32	[38]
MM Zeolite	CMC, Fe_3_O_4_	5	300	2.00	84	/	/	[25]
Na-Zeolite	NaCl	5	300	2.00	67	/	/	[25]
Zeolite-Mg	MgCl_2_	/	/	/	58	4.241	245.98	[26]
Biochar-MBC	MgCl_2_	5	200	0.40	829	3.210	2661.09	[28]
Biochar-MBC180	MgCl_2_	5	300	5.00	454	0.470	213.38	[39]
Biochar-MBC60	MgCl_2_	5	300	5.00	436	0.580	252.88	[39]
Biochar-MBC30	MgCl_2_	5	300	5.00	314	0.670	210.38	[39]
Biochar-MgBC	MgCl_2_	5	300	0.50	532	2.44	1298.08	[40]
Biochar-MBCW600	MgCl_2_	4	400	1.00	345	0.058	20.01	[41]
Zeolite-MZ	MgCl_2_, NaOH	5	1000	0.25	1656	0.578	957.17	This work

## Data Availability

The original contributions presented in this study are included in the article/Supplementary Materials. Further inquiries can be directed to the corresponding author.

## Supplementary Materials

The following supporting information can be downloaded at https://www.mdpi.com/article/10.3390/toxics14010085/s1. Figure S1: Fine XPS images of Pb 4f by Z-Pb and MZ-Pb; Table S1: Z-Pb and MZ-Pb EDS elemental composition [2,33,77,78,79,80,81,82,83,84,85,86,87,88,89].
