# Supplementary material for: Highly Efficient Adsorption of Pb(II) by Magnesium-Modified Zeolite: Performance and Mechanisms
Source: Toxics. 2026 Jan 17;14(1):85. doi: 10.3390/toxics14010085 (PMC12845599; doi:10.3390/toxics14010085)
Supplement: Supplementary file 1 [file toxics-14-00085-s001.zip › toxics-4062190-supplementary.pdf]

Supplementary data

Highly Efficient Adsorption of Pb(II) by Magnesium-Modified Zeolite: Performance and Mechanisms

Yuting Yang[1, †], Xiong Wang[1, †], Sumra Siddique Abbasi[1], Bin Zhou[2], Qing Huang[3], Shujuan Zhang[4], Xinsheng Xiao[1], Hao Li[1], Huayi Chen[1, *], Yueming Hu[1, *]

[1] School of Tropical Agriculture and Forestry, Hainan University, Haikou 570228, China
[2] Tropical Crops Genetic Resources Institute, Chinese Academy of Tropical Agricultural Sciences, Haikou 571101, China
[3] School of Environment Science and Engineering, Hainan University, Haikou 570228, China
[4] School of Architecture and Planning, Foshan University, Foshan 528000, China
[*] Correspondence: huayi93@hainanu.edu.cn (H.C.); 995149@hainanu.edu.cn (Y.H.)
[†] These authors contributed equally to this work.

To investigate the adsorption kinetics of Pb(II) on the residue, four kinetic models were used (Fu et al., 2024; Gao et al., 2025; Wang and Ariyanto, 2007; Xing et al., 2018), namely pseudo-first-order model, pseudo-second-order model, Elovich model and intraparticle diffusion model.

The pseudo-first-order equation (Chen et al., 2021; Shen et al., 2021; Wang and Ariyanto, 2007) can be represented as:

$$q_t = \mathrm{q}_e(1 - e^{-k_1 t})$$

where q_e and q_t are the amount of Pb(II) adsorbed on adsorbent (mg/g) at equilibrium and time t (h), respectively, and k_1 is the rate constants of the pseudo-first-order adsorption (h^{-1}), respectively.

The pseudo-second-order equation (Haider et al., 2025; Shen et al., 2021; Wang and Ariyanto, 2007) can be represented as:

$$q_t = \frac{\mathrm{t}}{\left(\frac{1}{k_2 {q_e}^2}\right) + \left(\frac{t}{\mathrm{q}_e}\right)}$$

where q_e and q_t are the amount of Pb(II) adsorbed on adsorbent (mg/g) at equilibrium and time t (h), respectively, and k_2 is the rate constants of the pseudo-second-order adsorption (mg/g h), respectively.

The Elovich equation (Haider et al., 2025; Tseng et al., 2022) can be represented as:

$$q_t = \left(\frac{1}{\beta_e}\right)\ln(\alpha_e \beta_e) + \left(\frac{1}{\beta_e}\right)\ln t$$

where q_e and q_t are the amount of Pb(II) adsorbed on the adsorbent (mg/g) at equilibrium and time t (h), respectively, α_e indicates the initial adsorption rate constant (mg/g·h), and β_e is the desorption rate constant (g/mg), respectively.

The intraparticle diffusion equation (Chen et al., 2021; Xiang et al., 2024; Xiong et al., 2011; M. Zhang et al., 2019) can be represented as:

$$q_t = K_{id}t^{0.5} + C$$

where q_t is the amount of Pb(II) adsorbed on the adsorbent (mg/g) at time t (h), K_{id} (mg/g $h^{0.5}$) is the intraparticle diffusion rate constant, and C is the boundary layer thickness, respectively.

The equilibrium isotherm curves were fitted using the Langmuir, Freundlich, and Temkin models (Fu et al., 2024; Xing et al., 2018).

The Langmuir equation (Chen et al., 2021; Haider et al., 2025; Wang and Ariyanto, 2007) can be represented as:

$$q_e = \frac{q_m k_L C_e}{1 + (k_L C_e)}$$

where q_e is the amount of Pb(II) adsorbed at equilibrium (mg/g), q_m is the maximum amount of Pb(II) adsorbed on adsorbent (mg/g), C_e is the equilibrium Pb(II) concentration (mg/L), and k_L is the Langmuir adsorption constant (L/mg), respectively.

The Freundlich equation (Haider et al., 2025; Li et al., 2005; M. Zhang et al., 2019) can be represented as:

$$q_e = k_F {C_e}^{1/n}$$

where q_e is the amount of Pb(II) adsorbed at equilibrium (mg/g), C_e is the concentration of Pb(II) in the solution (mg/L) at equilibrium, and k_F and n are the Freundlich constants related to adsorption capacity and adsorption intensity, respectively.

The Temkin equation (Mudhoo, 2025; Singh et al., 2023; X. Zhang et al., 2019) can be represented as:

$$q_e = B_1 \ln A + B_1 \ln{(C_e)}$$

where q_e is the amount of Pb(II) adsorbed at equilibrium (mg/g), C_e is the concentration of Pb(II) in the solution (mg/L) at equilibrium, B_1 stands for the Temkin constant, and A is the Temkin isotherm constant, respectively.

Table S1. Z-Pb and MZ-Pb EDS elemental composition.

Elements	MZ-Pb		Z-Pb	
	w%	atom%	w%	atom%
C	0.00	0.00	16.22	25.67
N	1.18	4.25	0.00	0.00
O	19.02	60.13	41.32	49.08
Na	0.58	1.28	0.08	0.07
Mg	4.5	9.38	0.24	0.19
Al	0.78	1.45	5.89	4.15
Si	3.41	6.14	27.17	18.39
K	0.05	0.06	2.33	1.13
Ca	0.10	0.13	1.82	0.86
Pb	70.38	17.18	4.91	0.45

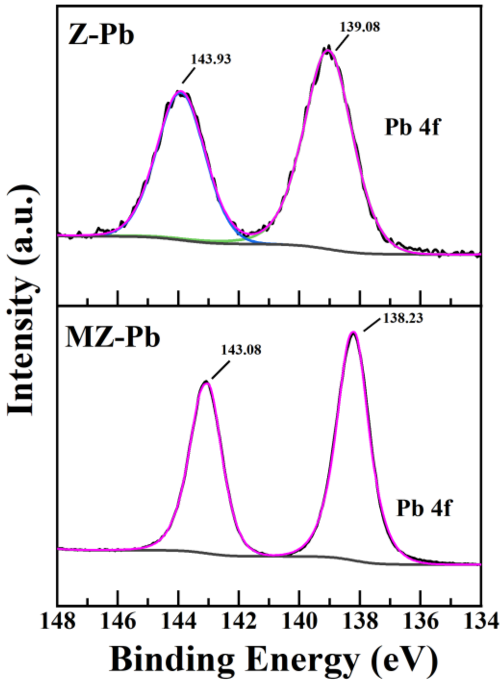

Figure S1. Fine XPS images of Pb 4f by Z-Pb and MZ-Pb.

References

Chen, Y., Tang, J., Wang, S., Zhang, L., Sun, W., 2021. Bimetallic coordination polymer for highly selective removal of Pb(II): Activation energy, isosteric heat of adsorption and adsorption mechanism. Chemical Engineering Journal 425, 131474. https://doi.org/10.1016/j.cej.2021.131474

Fu, T., Wu, S., Zhao, M., Zheng, X., Wang, Z., Jin, Z., Fan, C., 2024. Preparation and application of cattail residue-based magnetic cellulose composites for tetracycline antibiotics adsorption. Process Safety and Environmental Protection 189, 598–611. https://doi.org/10.1016/j.psep.2024.06.114

Gao, F., Tan, C., You, Y., Hou, Y., Yan, H., Lv, X., Dang, J., 2025. Efficient and selective adsorption of Ni(II) and Mo(VI) utilizing novel materials derived from in situ aminated Co/Zn-ZIF-Modified biochar. Separation and Purification Technology 359, 130669. https://doi.org/10.1016/j.seppur.2024.130669

Haider, B., Imran, M., Naeem, A., El-Beltagi, H.S., Arshad, H., Hussain, A., Zulfiqar, U., Rebouh, N.Y., Prasad, P.V.V., Djalovic, I., 2025. Adsorptive removal of lead from wastewater using pressmud with evaluation of kinetics and adsorption isotherms. Sci Rep 15, 22823. https://doi.org/10.1038/s41598-025-05169-9

Li, Y.-H., Di, Z., Ding, J., Wu, D., Luan, Z., Zhu, Y., 2005. Adsorption thermodynamic, kinetic and desorption studies of Pb2+ on carbon nanotubes. Water Research 39, 605–609. https://doi.org/10.1016/j.watres.2004.11.004

Mudhoo, A., 2025. Unveiling new insights: Revised Temkin adsorption isotherm parameters from fresh curve fits in adsorption studies. Chemical Engineering Science 311, 121585. https://doi.org/10.1016/j.ces.2025.121585

Shen, M., Song, B., Zeng, G., Zhang, Y., Teng, F., Zhou, C., 2021. Surfactant changes lead adsorption behaviors and mechanisms on microplastics. Chemical Engineering Journal 405, 126989. https://doi.org/10.1016/j.cej.2020.126989

Singh, S., U, B., Kumar Naik, T.S.S., Behera, S.K., Khan, N.A., Singh, J., Singh, L., Ramamurthy, P.C., 2023. Graphene oxide-based novel MOF nanohybrid for synergic removal of Pb (II) ions from aqueous solutions: Simulation and adsorption studies. Environmental Research 216, 114750. https://doi.org/10.1016/j.envres.2022.114750

Tseng, R.-L., Tran, H.N., Juang, R.-S., 2022. Revisiting temperature effect on the kinetics of liquid–phase adsorption by the Elovich equation: A simple tool for checking data reliability. Journal of the Taiwan Institute of Chemical Engineers 136, 104403. https://doi.org/10.1016/j.jtice.2022.104403

Wang, S., Ariyanto, E., 2007. Competitive adsorption of malachite green and Pb ions on natural zeolite. Journal of Colloid and Interface Science 314, 25–31. https://doi.org/10.1016/j.jcis.2007.05.032

Xiang, D., Zhu, R., Chen, Y., Zhu, M., Wang, S., Wu, Y., Luo, J., Fu, L., 2024. Preparation of amidoxime modified covalent organic framework for efficient adsorption of lead ions in aqueous solution. Chemical Engineering Journal 492, 152292. https://doi.org/10.1016/j.cej.2024.152292

Xing, P., Wang, C., Ma, B., Chen, Y., 2018. Removal of Pb(II) from aqueous solution using a new zeolite-type absorbent: Potassium ore leaching residue. Journal of Environmental Chemical Engineering 6, 7138–7143. https://doi.org/10.1016/j.jece.2018.11.003

Xiong, L., Chen, C., Chen, Q., Ni, J., 2011. Adsorption of Pb(II) and Cd(II) from aqueous solutions using titanate nanotubes prepared via hydrothermal method. Journal of

Hazardous Materials, Selected papers presented at the 2nd International Conference on Research Frontiers in Chalcogen Cycle Science and Technology, Delft, The Netherlands, May 31st-June 1st, 2010 189, 741–748. https://doi.org/10.1016/j.jhazmat.2011.03.006

Zhang, M., Zhu, L., He, C., Xu, X., Duan, Z., Liu, S., Song, M., Song, S., Shi, J., Li, Y., Cao, G., 2019. Adsorption performance and mechanisms of Pb(II), Cd(II), and Mn(II) removal by a β-cyclodextrin derivative. Environ Sci Pollut Res 26, 5094–5110. https://doi.org/10.1007/s11356-018-3989-4

Zhang, X., Li, Y., Hou, Y., 2019. Preparation of magnetic polyethylenimine lignin and its adsorption of Pb(II). International Journal of Biological Macromolecules 141, 1102–1110. https://doi.org/10.1016/j.ijbiomac.2019.09.061
